# Effect of organizational structure of the ICU on the prognosis: open format versus semi-closed format

**DOI:** 10.1186/cc12455

**Published:** 2013-03-19

**Authors:** T Taniguchi, M Okajima

**Affiliations:** 1 Kanazawa University Hospital, Kanazawa, Japan

## Introduction

In the year 2009, the organizational structure of the ICU in the Kanazawa University Hospital changed from an open to a semi-closed format ICU. The objective of this study was to evaluate the effect of this organizational change on outcome in high-risk surgical patients.

## Methods

The medical records of all consecutive high-risk surgical patients admitted to the ICU from 2006 to 2009 (open format, *n *= 1,598) and from 2009 to 2012 (semi-closed format, *n *= 1,521) were reviewed. Parameters studied were mortality and ICU length of stay.

## Results

Mortality of ICU patients was 9.9% in the open format group and 6.6% in the closed format group (*P *0.05). The average length of hospital stay was 4.9 days in the open format group and 4.8 days in the closed format group.

## Conclusion

Our results suggest that a semi-closed format is a more favorable setting than an open format to improve mortality in the ICU and to warrant safe outcome in this patient group.

